# Supplementary material for: Genomic and Transcriptomic Approaches Provide a Predictive Framework for Sesquiterpenes Biosynthesis in Desarmillaria tabescens CPCC 401429
Source: J Fungi (Basel). 2023 Apr 17;9(4):481. doi: 10.3390/jof9040481 (PMC10146329; doi:10.3390/jof9040481)
Supplement: Supplementary file 1 [file jof-09-00481-s001.zip › jof-2255069-supplementary.pdf]

Genomic and Transcriptomic Approaches Provide a Predictive Framework for Sesquiterpenes Biosynthesis in *Desarmillaria tabescens* CPCC 401429

Tao Zhang [1,*], Jianjv Feng [1], Wenni He [1], Xiaoting Rong [1], Hui Lv [1], Jun Li [1], Xinxin Li [1], Hao Wang [1], Lu Wang [1], Lixin Zhang [2,*] and Liyan Yu [1,*]

[1] Institute of Medicinal Biotechnology, Chinese Academy of Medical Sciences & Peking Union Medical College, Beijing 100050, China
[2] State Key Laboratory of Bioreactor Engineering, East China University of Science & Technology, Shanghai 200237, China
* Correspondence: zhangt218@hotmail.com (T.Z.); lxzhang@ecust.edu.cn (L.Z.); yly@cpcc.ac.cn (L.Y.)

TABLE OF CONTENTS

**Contents**	**Page**
Table S1. Summary statistics and STSs encoding genes of the sequenced *Armillaria* genomes. BUSCO scores indicate the completeness of genomic assemblies.	S3
Table S2. Gene distribution of encoding CAZymes in different mushroom.	S3
Table S3. List of upregulated DEGs coding glycosyl hydrolase family proteins.	S4-5
Table S4. List of upregulated DEGs coding transporter proteins.	S5-6
Table S5. List of upregulated DEGs coding fruiting body formation proteins.	S6
Table S6. List of upregulated DEGs coding SMs biosynthetic or related proteins.	S6-7
Figure S1. HPLC analysis of metabolite profiles obtained from the PDB, F1 and YMEG fermentation broth of *D. tabescens* CPCC 401429.	S8
Figures S2. RNA samples extracted from the *D. tabescens* CPCC 401429.	S8
Figure S3. Heatmap of three samples including PDB, F1, and YMEG fermentation broth obtained by RNA-seq of *D. tabescens* CPCC 401429.	S9
Figure S4. Heatmap of cytochrome P450s coding genes in three samples including PDB, F1, and YMEG fermentation broth obtained by RNA-seq of *D. tabescens* CPCC 401429.	S10
Sequences 1. *tef-1α* gene sequences of CPCC 401429 and sequenced *Armillaria* spp.	S10-12
Sequences 2. Codon optimized sequences of gene *DtSTS9* and *DtSTS10*.	S12

Table S1 Summary statistics and STSs encoding genes of the sequenced *Armillaria* genomes. BUSCO scores indicate the completeness of genomic assemblies.

**organisms**	**Assembly size (Mb)**	**GC%**	**BUSCO(%)**	**Number of genes**	**No. of STSs encoding genes**
D. tabescens CPCC 401429	50.36	47.14	92.4%	15,145	12
A. altimontana 837-10	73.74	47.8	-	-	13
A. borealis FPL87.14 v1.0	67.0	47.30	94.7%	21,969	9
A. cepistipes B5	75.52	47.71	97.9%	23,461	15
A. gallica 012m	87.31	47.38	99.8%	25,704	11
A. ostoyae C18/9	60.11	48.33	99.2%	22,705	12
A. fuscipes CMW2740	52.98	47.68	-	-	6
A. solidipes 28-4	55.74	48.26	99.8%	20,811	12
A. mellea DSM 3731	58.35	47.26	-	14,473	17

Table S2 Gene distribution of encoding CAZymes in different mushroom.

CAZymes	Total	GH	GT	AA	CE	PL	CBM
D. tabescens	509	199	52	145	78	20	15
A. gallica 012m	824	331	100	170	142	25	82
A. gallica Ar21-2	826	318	106	178	144	25	79
A. cepistipes B5	810	329	97	181	136	27	66
A. ostoyae C18/9	764	300	92	156	135	31	71
A. solidipes 28-4	789	302	93	158	150	29	79
A. bisporus	374	172	52	94	33	12	11
P. ostreatus	512	226	65	139	27	26	29
C. cinerea	475	188	72	129	47	18	21
L. edodes	449	241	71	86	32	11	14
S. commune	469	247	71	85	37	19	11
L. bicolor	302	151	73	53	9	7	9

Table S3 List of upregulated DEGs coding glycosyl hydrolase family proteins.

Gene ID	Gene function	Log_2 FC (F1/PDB)	Log_2 FC (YMEG/PDB)
gene00130	glycoside hydrolase/deacetylase	1.636756628	-0.24983613056
gene00131	rhamnogalacturonase A	4.665469633	0.403603111928
gene00135	glucose-6-phosphate 1-dehydrogenase	1.16434665	-0.402050248184
gene00175	family 9 glycosyl hydrolase	1.415373529	-1.43526141879
gene00229	glycoside hydrolase family 16 protein	1.074303532	-1.28005015301
gene00519	glycoside hydrolase family 74 protein	1.665210136	-0.16562418118
gene00520	oligoxyloglucan reducing cellobiohydrolase	2.273960444	0.966423593918
gene00541	probable beta-glucosidase L	1.451961391	-0.576197601669
gene00817	glycoside hydrolase family 28 protein	7.052520724	0.227696775757
gene01658	glycosylase	1.847567231	-0.224425714737
gene01670	glycoside hydrolase family 27 protein	1.479700705	1.833577335951
gene02382	pectate lyase	1.041890636	0.639574063733
gene02423	glycoside hydrolase family 16 protein	4.299574866	-0.359545773518
gene03225	glucoamylase	2.906825176	2.46158203245
gene03324	carbohydrate esterase family 9 protein	1.271900837	n.d.
gene03447	endoglucanase-4	1.273689527	-0.462099968279
gene03604	Ribose-phosphate pyrophosphokinase 2	1.040864385	2.3643945261582
gene03806	pectin lyase-like protein	1.505296299	-0.684519003802
gene04166	glycoside hydrolase family 71 protein	1.286959958	0.142683544611
gene04379	polysaccharide lyase family 4 protein	2.015460952	-0.971117686027
gene04526	alpha-mannosidase	1.632250566	n.d.
gene04696	carbohydrate esterase family 8 protein	1.498912527	n.d.
gene05077	six-hairpin glycosidase	1.601189581	0.438353901693
gene05090	glycoside hydrolase family 61 protein	1.833444422	n.d.
gene05226	endoglucanase V-like protein	5.404548172	0.47066171422
gene05485	glycosyl hydrolase family 61.5	3.000837207	1.544308808
gene05670	glycoside hydrolase family 31 protein	1.020283078	n.d.
gene05738	actin depolymerizing protein	1.15140835	0.589961832524
gene05851	glycoside hydrolase family 47 protein	1.152642492	-0.477325632583
gene06185	putative alpha-amylase	1.5216045	n.d.
gene06203	endopolygalacturonase 2 precursor	6.858761819	n.d.
gene06251	glycoside hydrolase family 3 protein	1.840956907	-2.15529261378
gene06654	chitin deacetylase 1	1.918996284	-0.226218783527
gene06655	carbohydrate-binding module family 1 protein	2.183041591	-0.0480090313808
gene06795	putative endo-xylogalacturonan hydrolase A	2.729302612	n.d.
gene07115	UDP-glucosyl transferase family protein	1.104721778	n.d.
gene07786	glycoside hydrolase family 76 protein	1.980960039	0.816473014841
gene08058	related to glucosidase I	1.442980532	0.737688716368
gene08221	glucoamylase	2.042472538	n.d.
gene10048	UDP-Glycosyltransferase/glycogen	1.712228706	-1.35174136141

	phosphorylase		
gene10174	D-xylulose 5-phosphate/D-fructose 6-phosphate phosphoketolase	3.291012092	-0.600961614303
gene10709	chitin deacetylase	3.285631957	0.161556650717
gene12159	glycoside hydrolase family 31 protein	1.020283078	0.296458040146
gene12854	glycosyl hydrolase	1.34702831	2.512871564
gene13258	endo-1,4-beta-xylanase F3	1.96373657	-2.07022390984
gene13557	related to endoglucanase c	1.755185551	-2.729915213
gene13739	arabinosidase	2.646105281	0.024530051578
gene14722	glycoside hydrolase family 5 protein	1.524154588	n.d.
gene15034	glycoside hydrolase family 16 protein	5.889010501	n.d.
gene15199	galactose mutarotase-like protein	1.459160227	n.d.

Note: n.d, not detected.

Table S4 List of upregulated DEGs coding transporter proteins.

Gene ID	Gene function	Log_2 FC (F1/PDB)	Log_2 FC (YMEG/PDB)
gene00481	OPT oligopeptide transporter	1.315189935	-1.67657778735
gene00524	amino acid permease	2.185697379	-0.223010432582
gene00834	MFS amino acid permease	1.003368506	-0.187934914113
gene01023	MFS general substrate transporter	1.560018823	-0.313974306574
gene01036	MFS general substrate transporter	2.577165695	n.d.
gene01134	SNQ2-ABC transporter	1.642307153	-1.80955609239
gene01539	solute symporter family transporter	1.305348845	-1.2174245891
gene01824	MFS general substrate transporter	1.000520889	n.d.
gene01859	hexose transporter	1.825479678	-0.0254543640102
gene03247	transport protein particle complex subunit	1.119939801	0.226832905239
gene03473	MFS monosaccharide transporter	2.243139826	-0.326748456729
gene03810	MFS general substrate transporter	2.230924629	-1.30308460336
gene03944	MFS polyamine transporter	2.482901816	0.171818896389
gene04550	putative efflux transporter	1.573662488	-0.212288143801
gene04627	MFS general substrate transporter	2.098731599	n.d.
gene04646	MFS general substrate transporter	1.603902816	1.85595869964
gene04659	MFS general substrate transporter	1.603902816	1.32958635746
gene05358	MFS general substrate transporter	1.850421432	-0.176487798557
gene05935	general substrate transporter	3.551250496	-1.0161930619
gene05943	general substrate transporter	2.001012795	n.d.
gene06166	auxin efflux carrier	1.670233934	-0.520980883513
gene06180	MFS general substrate transporter	1.662828577	-2.01602477653
gene06524	MFS general substrate transporter	1.223765827	n.d.
gene06585	MFS general substrate transporter	1.267488912	-0.200056674124
gene06739	drug: H+ antiporter	3.503668675	-0.0322001201191
gene07162	related to sugar transport protein STP1	1.238781649	-0.0565513942988
gene07723	MFS general substrate transporter	1.894546831	n.d.
gene09360	MFS quinate transporter QutD	1.160447189	-1.46948239886

gene09452	general substrate transporter	1.481599665	n.d.
gene09983	probable ABC1 transport protein	6.48375099	-1.14141533
gene10135	MFS general substrate transporter	1.641874348	-3.51779830611
gene10495	vacuolar amino acid permease	1.465755153	-0.485536693732
gene11126	MFS general substrate transporter	3.274520412	n.d.
gene11655	MFS general substrate transporter	1.042300098	-3.83093668498
gene11789	related to sugar transporter STL1	1.226849589	-2.02504197514
gene12821	sodium/hydrogen exchanger	1.011451095	-0.0789032760406
gene12938	major facilitator superfamily MFS-1	1.2687358	-0.259974067384
gene13044	MFS general substrate transporter	1.059909857	4.20185185
gene13210	MFS general substrate transporter	1.290812977	-0.380129337395
gene13907	ABC transporter	1.172319797	1.929918115
gene13947	amino acid transporter	1.278509527	-1.652118207
gene13949	probable ABC1 transport protein	1.071407599	-0.59830699228
gene14251	MFS transporter	3.718459472	n.d.
gene14269	MFS general substrate transporter	1.573732731	0.529174012607
gene14271	MFS general substrate transporter	1.541892783	1.158300235
gene14844	ABC transporter	1.471831588	n.d.

Note: n.d, not detected.

Table S5 List of upregulated DEGs coding fruiting body formation proteins.

Gene ID	Gene function	Log_2 FC (F1/PDB)	Log_2 FC (YMEG/PDB)
gene04243	related to Fruiting body protein SC3	5.340951801	0.594373716203
gene04245	fungal hydrophobin	3.82962318	0.989459600761
gene04246	related to Fruiting body protein SC1	2.506056434	-0.751128867326
gene04247	hydrophobin-domain-containing protein	2.105011016	0.0779214304858
gene04324	fungal hydrophobin	4.160970852	n.d.
gene04692	hydrophobin-251	6.831734789	-1.696259492
gene04697	related to Hydrophobin-3	4.307517909	0.153202922897
gene04699	related to Hydrophobin-3	7.754287488	-1.604504295
gene04706	hydrophobin-263	7.778137784	-0.206645682457
gene04710	hydrophobin-domain-containing protein	9.852301707	2.117760549
gene04712	fungal hydrophobin	8.724713452	-5.83793974
gene04715	type 1 hydrophobin	2.92780199	1.113142042
gene04718	type 1 hydrophobin	2.92780199	-0.921029753151
gene04721	fungal hydrophobin	8.724713452	2.157708128
gene04723	hydrophobin-domain-containing protein	9.852301707	-1.64757255525
gene04914	related to Fruiting body protein SC1	5.150848003	-0.373881660221
gene04915	fungal hydrophobin	1.4075292	1.151989125
gene07174	hydrophobin	1.388678251	-0.970695756838

Note: n.d, not detected.

Table S6 List of upregulated DEGs coding SMs biosynthetic or related proteins.

Gene ID	Gene function	Log_2 FC (F1/PDB)	Log_2 FC (YMEG/PDB)
gene00493	acetyl-CoA synthetase-like protein	3.065823293	0.218816302527

gene01032	acetyl-CoA synthetase-like protein	5.555017062	n.d.
gene01869	related to fusarubin cluster-monooxygenase	2.329064949	1.011694285
gene02330	acetyl-CoA synthetase-like protein	3.541431146	-0.638692151918
gene02835	uncharacterized protein ARMOST_10340	1.085551392	0.00170375424054
gene02836	induced alcohol dehydrogenase Bli-4	4.026315383	-0.699697933376
gene02837	NAD(P)-binding protein	2.067797132	n.d.
gene02838	Orsellinic acid synthase ArmB	2.514735172	n.d.
gene02842	uncharacterized protein ARMOST_13668	2.281776349	1.346017439
gene02843	alcohol oxidase	2.702336574	1.424562353
gene02844	O-methylsterigmatocystin oxidoreductase	4.301261384	-0.713820992469
gene02845	terpenoid synthase	3.864488643	n.d.
gene02846	cytochrome P450	7.930682994	0.992385846421
gene02853	cytochrome P450	8.427293813	-3.127413831
gene04818	related to coenzyme a synthetase	2.499394555	0.34610485054
gene07626	terpene synthase	1.394308283	0.0301949917736
gene08450	putative polyketide synthase	3.597752426	0.573053316724
gene08841	anthranilate synthase component 2	1.246161844	-0.507713697504
gene11200	clavaminate synthase-like protein	2.486362036	1.466150257
gene11245	thiazole biosynthetic enzyme	3.612534404	0.305425494396
gene12094	clavaminate synthase-like protein	3.40409459	1.48291766
gene12096	clavaminate synthase-like protein	3.156497267	0.286457153163
gene12939	alpha-muurolene synthase	1.978606026	-0.650620180304

Note: n.d, not detected.

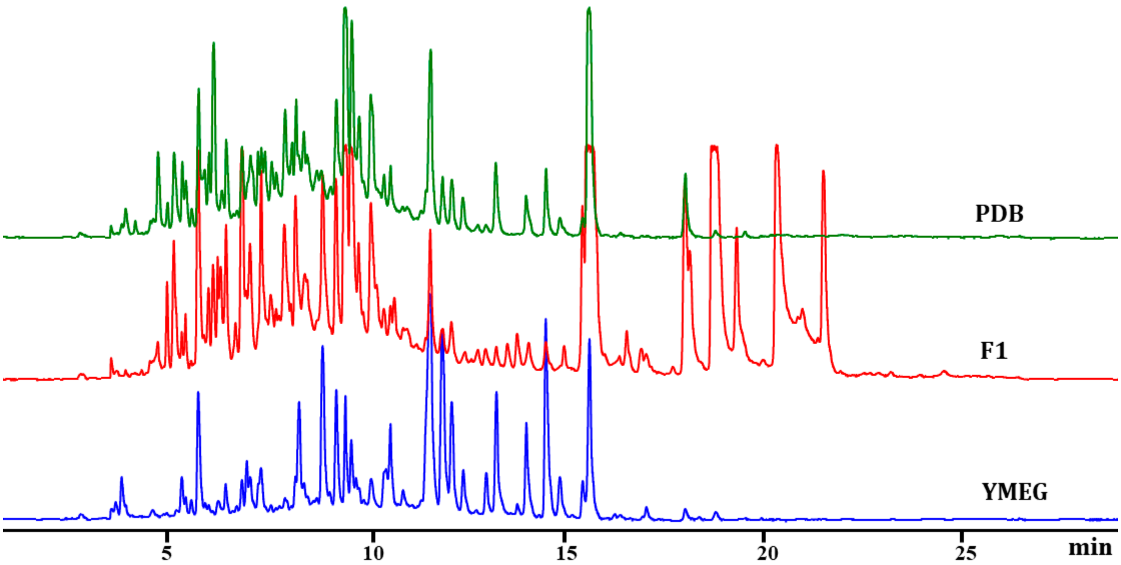

Figure S1. HPLC analysis of metabolite profiles obtained from the PDB, F1 and YMEG fermentation broth of *D. tabescens* CPCC 401429. The three samples including PDB, F1, and YMEG broth with *Armillaria* cultures were extracted with ethyl acetate two times. Each extract was prepared and analyzed according to the conditions described elsewhere.

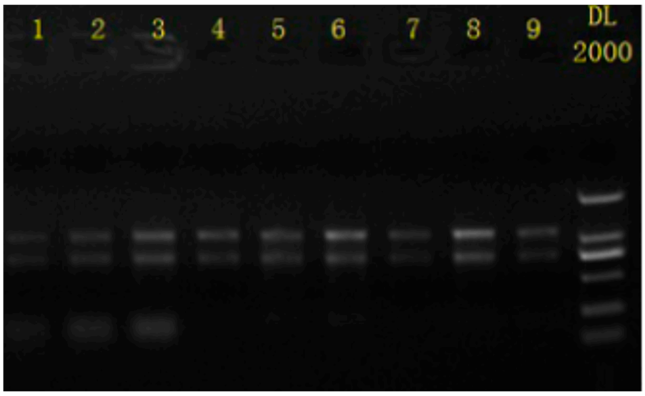

Figure S2. RNA samples extracted from the *D. tabescens* CPCC 401429. M, DNA marker; Lanes 1, 2, and 3 are RNA samples isolated from PDB broth; Lanes 4, 5, and 6 are RNA samples isolated from F1 broth; Lanes 7, 8, and 9 are RNA samples isolated from YMEG broth.

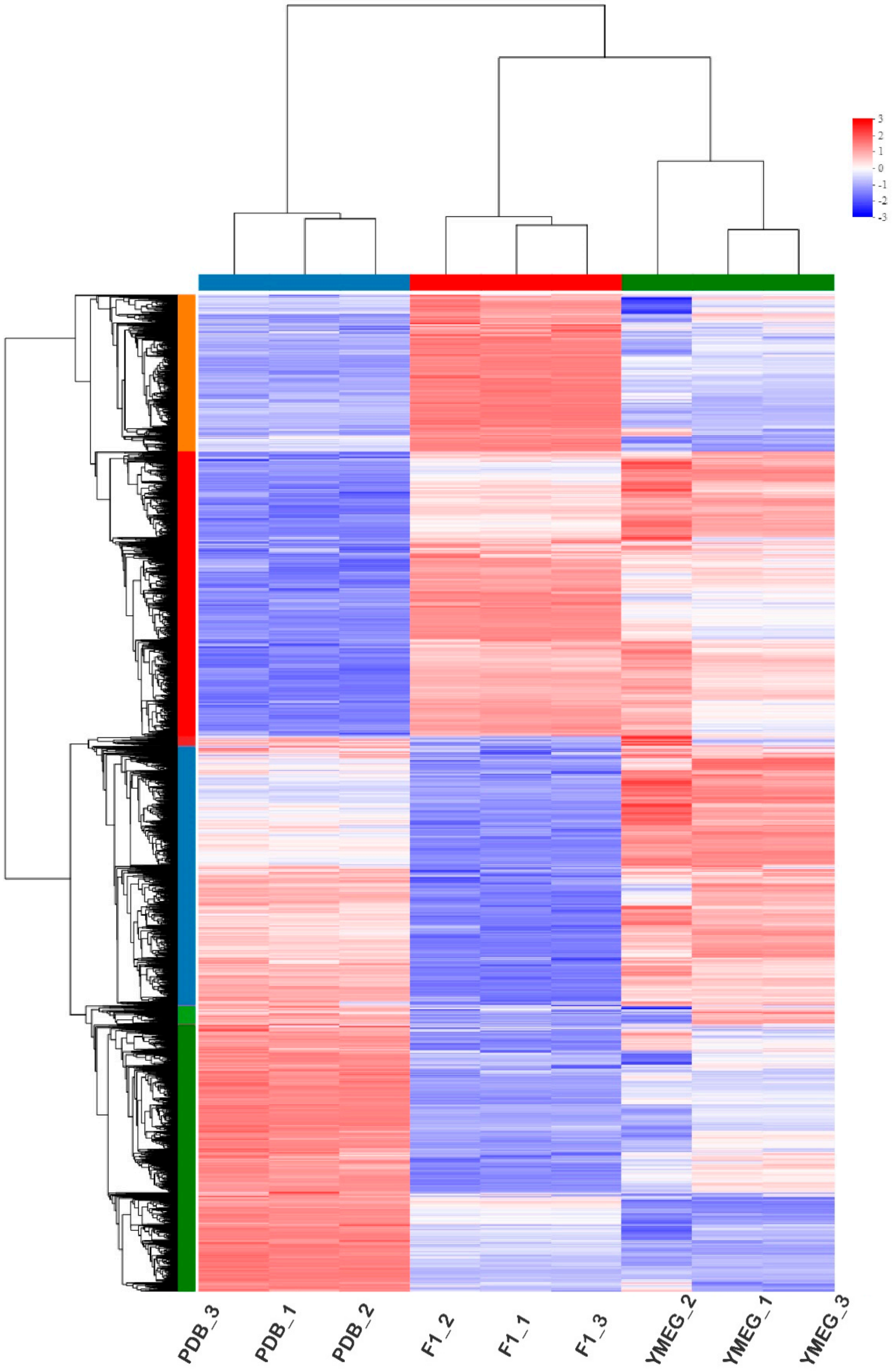

Figure S3. Heatmap of three samples including PDB, F1, and YMEG fermentation broth obtained by RNA-seq of *D. tabescens* CPCC 401429. PDB-1, PDB-2, and PDB-3 are samples of PDB fermentation broth; F1-1, F1-2, and F1-3 are samples of F1 fermentation broth; YMEG-1, YMEG-2, and YMEG-3 are samples of YMEG fermentation broth.

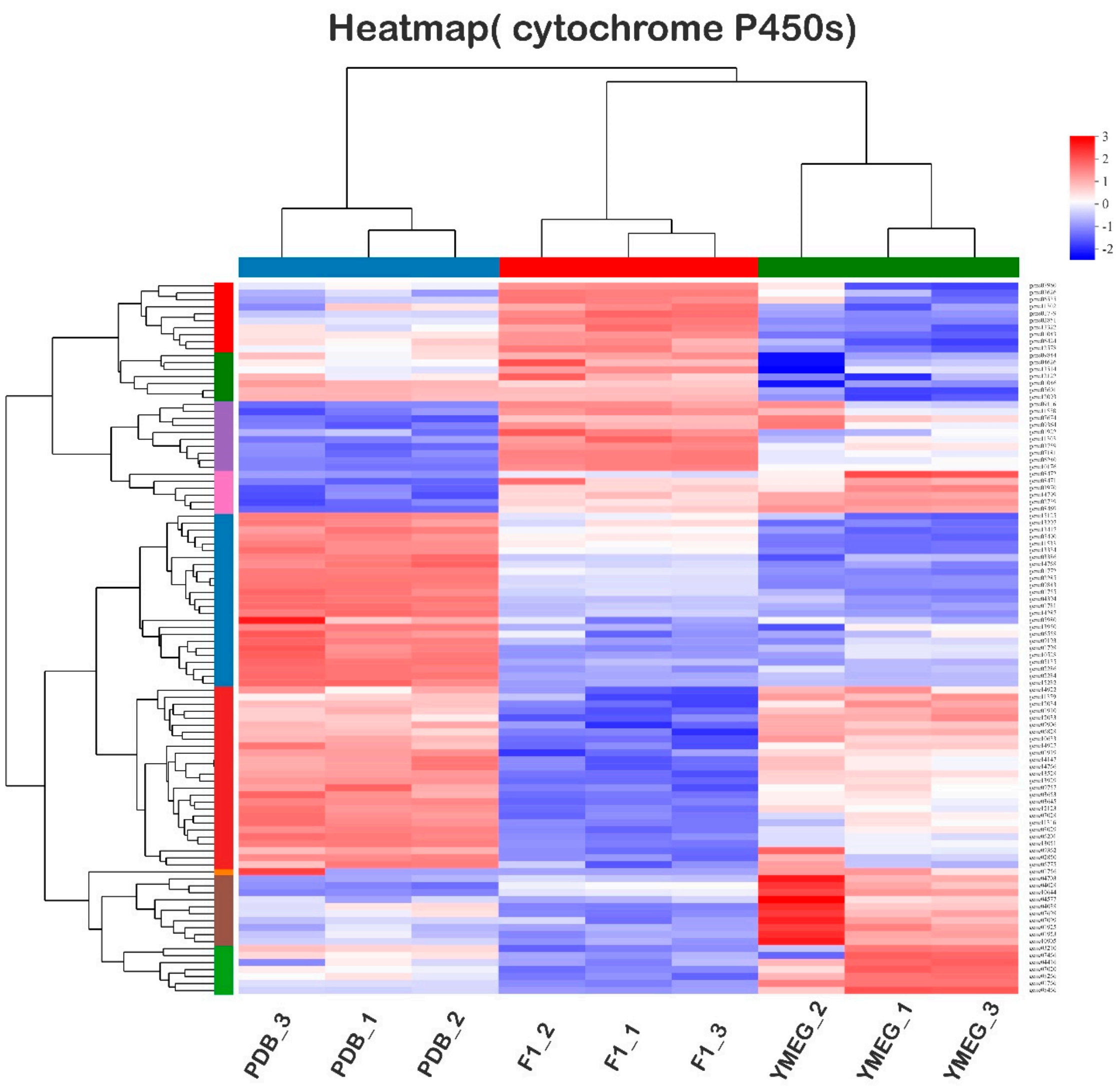

Figure S4. Heatmap of cytochrome P450s coding genes in three samples including PDB, F1, and YMEG fermentation broth obtained by RNA-seq of *D. tabescens* CPCC 401429. PDB-1, PDB-2, and PDB-3 are samples of PDB fermentation broth; F1-1, F1-2, and F1-3 are samples of F1 fermentation broth; YMEG-1, YMEG-2, and YMEG-3 are samples of YMEG fermentation broth.

Sequences 1. *tef-1α* gene sequences of CPCC 401429 and sequenced *Armillaria* spp.

> ***tef-1α* gene (*Desarmillaria tabescens* CPCC 401429)**

AGAACATGATCACTGGTACCTCCCAAGCTGATTGTGCCATCCTTATCATTGCTGGTGGTACTGGTGAAT
TCGAAGCCGGTATCTCCAAGGATGGTCAGACCCGAGAGCACGCCCTTCTTGCCTTCACTCTTGGTGTC
AGGCAGCTCATTGTTGCCGTCAACAAGATGGACACCACCAAGGTACGAACCCTACCCCATCGCTTTTT
CTTTCGCGAAGTCCGACATTTGTCTTAGTGGAGTGAGGACCGATTCAACGAAATCGTCAAGGAAACCT
CCACCTTCATCAAGAAGGTCGGCTACAACCCCAAATCTGTTGCTTTCGTCCCCATCTCTGGATGGCACG
GTGACAACATGTTGGAGGAGTCCGCCAAGTAAGTCATTACCATATTATGAGCGATGCGGCGGCTTAAC
GTTATTGAAAGCATGCCATGGTACAAGGGCTGGACGAAGGAGACCAAGGCCGGTGTCGTCAAGGGCA
AGACTCTCCTCGATGCTATTGATGCCATTGAGCCCCCTGTCCGACCCTCCGACAAGCCTCTCCGTCTTC
CTCTCCAGGATGTCTACAAAAT
//

> **tef-1α gene (*Armillaria cepistipes* B5)**

AGAACATGATCACCGGTACCTCCCAGGCTGATTGTGCCATTCTCATTATCGCTGGTGGAACTGGTGAGT
TCGAAGCCGGTATCTCCAAGGACGGTCAGACCCGAGAGCACGCCCTCCTTGCCTTCACCCTCGGTGTC
AGGCAGCTCATTGTCGCTGTCAACAAGATGGACACCACCAAGGTACGAGATCTACTGTTTTACTTTTTT

CCTTAGGCATATCTGACTGGTATCTCAGTGGAGCGAGGACCGGTTCAACGAAATCGTCAAGGAAACCT
CCACCTTCATCAAGAAGGTCGGCTACAACCCCAAGGCCGTTGCGTTCGTCCCCATCTCTGGATGGCAC
GGTGATAACATGTTGGAGGAGTCCCCCAAGTAAGTCCCTTACCCAACTATGACCAGGGCTGCTTCTTA
ATGTTCTCTATAGCATGCCATGGTACAAGGGCTGGACCAAGGAGACCAAGGCCGGTGTCGTCAAGGGC
AAGACTCTCCTCGATGCCATTGACGCCATTGAGCCCCCTGTCCGTCCCTCCGACAAGCCTCTCCGTCTC
CCTCTCCAGGATGTCTACAAAAT
//
> **tef-1α gene (*A. fuscipes* CMW2740)**
AGAACATGATCACCGGTACCTCCCAGGCTGATTGTGCCATTCTCATCATCGCTGGTGGAACTGGTGAGT
TCGAAGCCGGTATCTCCAAGGACGGACAGACCCGAGAGCATGCCCTCCTTGCCTTCACCCTCGGTGTC
AGGCAGCTTATCGTTGCCGTCAACAAGATGGACACTACTAAGGTACACGATTCTCTGTTTCATCCTTTC
TTTGGCTAATCTCATTTTGATTTTAGTGGAGCGAGGACCGATTCAACGAAATTGTCAAGGAAACCTCTA
CCTTCATCAAGAAGGTCGGCTATAACCCCAAGGCTGTCGCTTTCGTCCCCATCTCTGGATGGCACGGTG
ATAACATGTTGGAGGAGTCCGTCAAGTAAGCTTACATCCGACTATGATCTATGATTAATGGTAGATCTTG
ACCTTCTCTGTAGCATGCCCTGGTACAAGGGCTGGACCAAGGAGACCAAGGCCGGTGTCGTCAAGGG
CAAGACTCTCCTTGATGCCATCGACGCTATTGAGCCCCCTGTTCGTCCCTCCGACAAGCCTCTCCGTCT
CCCTCTCCAGGATGTTTACAAAAT
//
> **tef-1α gene (*A. gallica* 012m)**
AGAACATGATCACTGGTACCTCCCAGGCTGATTGTGCCATCCTCATCATCGCTGGTGGAACTGGTGAGT
TCGAGGCCGGTATCTCCAAGGACGGTCAGACCCGAGAGCACGCCCTCCTTGCCTTCACCCTCGGTGTC
AGGCAGCTCATTGTCGCCGTCAACAAGATGGACACCACCAAGGTACGAGATCTGCTGCTTTGCCTTTT
GTTTAGCCAAATCTGACTGTTATCTCAGTGGAGTGAGGACCGGTTCAACGAAATCGTCAAGGAAACCT
CTACCTTCATCAAGAAGGTCGGCTACAACCCCAAGGCCGTTGCTTTCGTCCCCATCTCTGGATGGCAC
GGTGACAACATGTTGGAGGAGTCCGCCAAGTAAGTCTTTACCCAGCTATGATCAGTGCTGTTTCTTAAC
GTTCTCTGTAGCATGCCATGGTACAAGGGCTGGACCAAGGAGACCAAGGCTGGTGTCGTCAAGGGCA
AGACTCTCCTCGATGCCATTGACGCCATTGAGCCCCCTGTCCGTCCCTCCGACAAGCCTCTCCGTCTCC
CTCTCCAGGATGTCTACAAAAT
//
> **tef-1α gene (*A. gallica* Ar21-2)**
AGAACATGATCACCGGTACCTCCCAGGCTGATTGTGCCATTCTCATCATCGCTGGTGGAACTGGTGAGT
TCGAAGCTGGTATCTCCAAGGACGGTCAGACCCGAGAACACGCCCTCCTTGCCTTCACCCTCGGTGTC
AGGCAGCTCATTGTCGCCGTCAACAAGATGGACACCACCAAGGTACGAGATCTGTTGCTTTGCCTTGT
GTTTAGCCAAATCTAACTGTTATCTCAGTGGAGCGAGGACCGGTTCAACGAAATTGTCAAGGAAACCT
CCACCTTCATCAAGAAGGTCGGCTACAACCCCAAGGCCGTTGCTTTCGTCCCCATCTCTGGATGGCAC
GGTGATAACATGTTGGAGGAGTCCGCCAAGTAAGTCTTTACCCAACTATGATCAGTGCTGCCTCTTAAC
GTGTTTTGTAGCATGCCATGGTACAAGGGCTGGACCAAGGAGACCAAGGCCGGTGTCGTTAAGGGCA
AGACTCTCCTCGATGCCATTGACGCCATTGAGCCCCCTGTCCGTCCCTCCGACAAGCCTCTCCGTCTCC
CTCTCCAGGATGTCTACAAAAT
//
> **tef-1α gene (*A. mellea* DSM 3731)**
AGAACATGATCACCGGTACCTCCCAGGCTGATTGTGCCATTCTCATCATCGCTGGTGGAACTGGTGAGT
TCGAAGCCGGTATCTCCAAGGACGGGCAGACCCGAGAGCACGCCCTCCTTGCCTTCACCCTCGGTGT
CAGGCAGCTCATTGTCGCCGTCAACAAGATGGACACCACCAAGGTACAGGATCTGCTGTTTCAGTTTT
TCTTTAGTCAAATCTGATTGTTATCTCAGTGGAGCGAGGACCGATTCAACGAAATCGTCAAGGAAACC
TCTACCTTCATCAAGAAGGTCGGTTACAACCCCAAGGCTGTTGCTTTCGTCCCCATCTCTGGATGGCAC
GGTGATAACATGTTGGAGGAGTCCGCCAAGTACGTCCTTACTTAACTATGATCCGTACTGAGTCTTAAC
GTTCTCTGTAGCATGCCATGGTACAAGGGCTGGACCAAGGAGAGTAAGGCTGGTGTCGCCAAAGGCA
AGACTCTCCTCGATGCCATTGACGCCATTGAACCCCCTGTTCGTCCCTCCGACAAGCCTCTCCGTCTTC
CTCTCCAGGATGTCTACAAAAT
//
> **tef-1α gene (*A. ostoyae* C18/9)**
AGAACATGATCACTGGTACCTCCCAGGCTGATTGTGCCATTCTCATCATCGCTGGTGGAACTGGTGAAT
TTGAAGCCGGTATTTCCAAGGACGGCCAGACTCGAGAGCACGCCCTCCTTGCCTTCACCCTTGGTGTC
AGGCAGCTCATCGTCGCCGTCAACAAGATGGACACCACCAAGGTACGAGATCTATCGTTTTACCTTTTA
CCTTAGGCAAATCTGACTGTTATCTCAGTGGAGCGAGGACCGGTTCAACGAAATCGTCAAGGAAACTT
CCACCTTCATCAAGAAGGTCGGCTACAACCCCAAGGCCGTTGCTTTCGTCCCCATCTCTGGATGGCAC

GGTGATAACATGTTGGAGGAGTCCGCCAAGTAAGTCCTTACCTAAGTATGACCAGTGCTGCCTCTTAAC
GTTCTCTGTAGCATGCCATGGTACAAGGGCTGGACCAAGGAGACCAAGGCCGGTGTCGTCAAGGGCA
AGACTCTCCTCGATGCCATTGACGCCATTGAGCCCCCTGTCCGTCCCTCCGACAAGCCTCTCCGTCTCC
CTCTCCAGGATGTCTACAAAAT
//

> **tef-1α gene (*A. solidipes* 28-4)**

AGAACATGATCACTGGTACCTCCCAGGCTGATTGTGCCATTCTCATCATCGCTGGTGGAACTGGTGAGT
TCGAAGCCGGTATTTCCAAGGACGGCCAGACCCGAGAGCATGCCCTCCTTGCCTTCACCCTCGGTGTC
AGGCAGCTCATCGTCGCCGTCAACAAAATGGACACCACCAAGGTACGAGATCTACTGTTTTACCTTTT
TCCTTAGGCAAATCTGACTGTCATCTCAGTGGAGCGAGGACCGGTTCAACGAAATCGTCAAGGAAAC
CTCCACCTTCATCAAGAAGGTCGGCTACAACCCCAAGGCTGTTGCTTTCGTCCCCATCTCTGGATGGC
ACGGTGATAACATGTTGGAGGAATCCGCCAAGTAAGTCCCTTACCCAACTATGACCAGTGCTGGCTCT
TAACGTGCTCTGTAGTATGCCATGGTACAAGGGCTGGACCAAGGAGACTAAGGCTGGTGTCGTCAAGG
GCAAGACTCTCCTCGATGCCATTGACGCCATTGAGCCCCCTGTCCGTCCCTCCGACAAGCCTCTCCGT
CTCCCTCTCCAGGATGTCTACAAAAT
//

> **tef-1α gene (*A. altimontana* 837-10)**

TTCATCAAGAACATGATCACCGGTACCTCCCAGGCTGATTGTGCTATTCTCATCATCGCTGGTGGAACT
GGTGAGTTCGAAGCCGGTATCTCCAAGGACGGTCAGACCCGAGAGCACGCCCTCCTTGCCTTCACCC
TCGGTGTCAGGCAGCTCATTGTCGCCGTCAACAAGATGGACACCACCAAGGTATGAGATCTCTGCTTT
GCCTTTTGTTTAGCCAAATCTGACTGTTATCTCAGTGGAGCGAGGACCGGTTCAACGAAATCGTCAAG
GAAACCTCCACCTTCATCAAGAAGGTCGGCTACAACCCCAAGGCTGTTGCTTTCGTTCCCATCTCTGG
ATGGCACGGTGATAACATGTTGGAGGAGTCCGCTAAGTAAGTCTTTACCCAACTATGATCAGTACTGCC
TCTTAACGTTCTCTGTAGCATGCCATGGTACAAGGGCTGGACCAGGGATACCAAGGCCGGTGCCGTCA
AGGGTAAGACTCTCCTCGATGCCATTGACGCCATTGAGCCCCCTGTCCGTCCCTCCGACAAGCCTCTC
CGTCTCCCTCTCCAGGATGTCTACAAAAT
//

Sequences 2. Codon optimized sequences of gene *DtSTS9* and *DtSTS10*.

>***DtSTS9***

ATGACTCTATCTACAGCAACCAGACCTACTAGCACCTACTCATTAGCTGGCAGGCTTAAAACTCGTAAA
CCAAAGTCAGCTTCCGAAGAAATAGGTGATACTATAAGGGCGTTCCTACACCATTTCGACACTACAAA
CCTTCCTTCAATCTGGGATCCCACCTTAGAAGCACTATGCTTAGCAGAGGCAAAAAGAAGAGGATACA
GCATAGATGTCTTAAGACCATATTTAACCTTGGGTCTAAACATTACAGTTTCTGCTTACCATCATATTGAC
AACGCAAACGTAAAGGTCTTTATTGCTTTCTTTTGTATGTTTTTGATATATTTAGATGATGTATACCCGGA
CGATAGTGATGCATTAGGGGGAGTTCCAGATTTTACCCGTCACTTCACGTCTAGCGAAAAGCAATCTTC
AAAGATCCTTAACGACTTAGCTGATTTGTTAACGGAATCTTCTCAACACTTTGGTAAGGTCACAGCGG
ACTTTATTGTTCACTCTGCTCTGAAGTTTATTACAGGGCTAATATTAGAGGTGCAAAGCAAGCAAGAAA
GCACCCAGAGAGTTGAAGAATACGCCATTTTCTTGAGAGAGCAATGTGGATTGGCTGAAGCATTCGCT
ATGTTTATCTTCCCAAGAGACGTTCCTTTCCATCTGTATATCCAAGCTTTGCCTTCTATGAGAGATTTTAT
AGATTTCGCGAACGACATACTTTCTTTCTACAAGGAAGAATGTTCTGGAGATACTAACAATTTCATTTC
CCTACTAGCACAGGCTAGATCTGCACCAAAGAACCAAGTGTTAAGGTACGTAGCAACACAATGTTTAG
AGTCCTATGAACGTGCTTTGAGAATTTTGTCTCCACATCAAGAGGCCTATTTAGCTTTTGAAGAATTTA
CCGACGGATACCTAGCCTTACATTTAGGCATGAAGAGATACAGGTTGTCAGAACTGGGCTAG
//

> ***DtSTS10***

ATGACTTTAAGCACCTCAACGACAAGAACAAGAAGATGTATTAGACGTAGAGATATGCCATCCTGGAC
ATCTGCATTATCAAGTTTAGCAGGTCACTTGTTGGCTTTGAAACCAAGACATGCTAGCGAAGAAGTAG
GTGATACAATACTGTCCTTTTTAGACCATTTCGACACCTCCAACTTGCCATCTATTTGGGACCCGTCATT
GCAAGCTTTATGTCTAGTAGAAGCTGAAAGACGTGGTTACTCCGTAGAAGTGTTAAAGCCTTACTTAGT
CTTGGGATTGAACATAACAACTGCTGCGTATCATTACATCGACGATACAAACGTTAAAATGTTCATTTCT
TTCTTTTGTATGTTCCTAATATACTTGGACGACGTTTACCCAGACGATTCTGATGCATTAGGGGGTGTTC
CAGACTTCACCAGGAGATTCACATCTTCCGAAAAACAGTCCTCAAAGATTCTGAATGACTTAGCTGAC
TTATTGACAGAGTCTTCACAACACTTCGGAAAAGTTACTGCCGATTTCATCGTTCATAGTGCTTTAAAG
TTTATTACTGGTTTAATATTGGAAGTGCAATCTAAGTTCGAACCCACTCATAGAATAGAAGAATACGCCA
TCTTTTTGCGTGAACAGTGTGGTTTAGCTGAAGCTTTCGCCATGTTTATTTTCCCGAGGGACATCCCGT

ACCACTTATATATTCAGGCCTTGCCATCAATGAGAGATTTCATAGACTTTGCTAATGACATTTTGTCTTTC
TACAAAGAAGAATGCTCTGGCGATACAAATAATTTCATCTCATTACTAGCACAAGCGAGAGGTTCCCCA
AAGATTAAGGTATTACGTTACGTAGTCAGGCAGTGTCTAACTGCATATGAAAGAACTTTGAGAATTTTG
TCCCCTCATAGAGAAGCCCATAAGGCGTTTCGTAATTTCACAAACGGTTATTTAGCATTGCACTTGGGA
ATGAAGAGGTATAGGCTTTCAGAGCTTGGCTAA
//
